# A novel phenotype-based drug-induced liver injury causality assessment tool (DILI-CAT) allows for signal confirmation in early drug development

**DOI:** 10.1111/apt.16836

**Published:** 2022-03-09

**Authors:** Richard P. Hermann, Don C. Rockey, Ayako Suzuki, Michael Merz, Hans L. Tillmann

**Affiliations:** 1 AstraZeneca, Gaithersburg, Maryland, USA; 2 Digestive Disease Research Center, Medical University South Carolina, Charleston, South Carolina, USA; 3 Duke University Medical Center, Durham, North Carolina, USA; 4 Durham VA Medical Center, Durham, North Carolina, USA; 5 AstraZeneca, Freiburg, Germany; 6 Division of Gastroenterology, Hepatology & Nutrition, East Carolina University, Greenville, North Carolina, USA; 7 Greenville VA Health Care Center, Greenville, North Carolina, USA

## Abstract

**Background:**

Drug-induced liver injury (DILI) requires accurate case adjudication, with expert opinion being the current best practice.

**Aim:**

We utilised a novel DILI causality assessment tool (DILI-CAT), which uses drug-specific liver injury phenotypes, to examine potential DILI in early phase ximelagatran clinical development.

**Methods:**

We conducted a retrospective analysis of liver injury events from four *S*troke *P*revention using an *OR*al *T*hrombin *I*nhibitor in Atrial Fibrillation (SPORTIF) trials, in which patients were randomised to receive oral ximelagatran or adjusted-dose warfarin. A stepwise process was used with iterative adjustments. The DILI phenotype was characterised by latency, *R*-value, and AST/ALT ratio. A scoring algorithm was applied to liver events to assess how closely the liver events matched the Interquatile-Range for the working phenotype for each of the three parameters.

**Findings:**

Data from 3115 patients included in the SPORTIF trials as above were available. The initial ximelagatran phenotype was developed based on five liver injury cases from the ximelagatran arm and was then validated against an additional eight cases (5 ximelagatran, 3 warfarin); in these eight cases, there was a statistically significant difference in the total DILI-CAT scores of the two drugs (*p* = 0.016) between ximelagatran and warfarin. Together, these ten ximelagatran cases generated a second, refined ximelagatran phenotype, which was validated against an additional 75 cases (53 ximelagatran/22 warfarin)–again with statistically significant different DILI-CAT ximelagatran vs. warfarin scores (*p* < 0.001).

**Conclusion:**

DILI-CAT, a clinically intuitive, data-driven, computer-assisted scoring algorithm, is a useful tool for early detection of drug’s hepatotoxicity in clinical drug development.

## Graphical Abstract

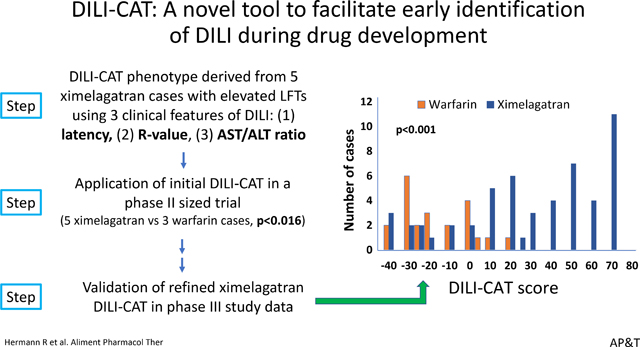

## 1 | INTRODUCTION

Drug-induced liver injury (DILI) is a serious safety concern during drug development. A critical issue in DILI diagnosis is its accurate adjudication. A number of quantitative and semi-quantitative assessment tools have been developed.^1,2^ The most widely used tool, the Roussel Uclaf Causality Assessment Method (RUCAM), has been in use for almost three decades; however, this tool and the other tools do not consider a drug’s signature (also known as a drug’s phenotypes) characteristic for a specific drug. This point is emphasised by the fact that expert opinion,^3^ which by its nature is likely to incorporate drug phenotypes into the adjudication process, appears to be superior to RUCAM in DILI adjudication.^4–6^ Experts likely intuitively take into account drugs’ DILI phenotypes in adjudication process even when no formalised phenotype has been developed. Therefore, many experts recommend expert opinion as the optimal adjudication process.^7^

Here, we hypothesised that a DILI causality assessment tool (DILI-CAT), as described earlier,^8,9^ can be used to help clinically determine the causality of DILI throughout a clinical development programme, starting in the early phases, by recognition of drug-specific phenotypes and application of a data-driven scoring algorithm to enhance the detection of liver signals and their adjudication during the drug development process.

In 2006, after reports of hepatotoxicity, the US Food and Drug Administration (FDA) halted clinical development of ximelagatran, an oral direct thrombin inhibitor designed for the prevention or treatment of thromboembolic conditions, due to concerns about DILI. Therefore, we have taken advantage of the extensive data set available from this programme to explore the possibility that the DILI-CAT could identify a drug-specific DILI signature early in development. Using ximelagatran clinical trial data, we describe the clinical signature of ximelagatran-associated liver injury with an aim to optimise accurate signal detection of true ‘liver signals’ using the DILI-CAT.

## 2 | METHODS

### 2.1 | Data source

Patients enrolled in the four *S*troke *P*revention using an *OR*al *T*hrombin *I*nhibitor in Atrial *F*ibrillation (SPORTIF) trials had a diagnosis of atrial fibrillation and had a planned treatment duration of >35 days. All four trials randomised patients to receive oral ximelagatran or adjusted-dose warfarin. The phase 2 trials included SPORTIF 2 and SPORTIF 4, and the phase 3 trials included SPORTIF 3 (open-label) and SPORTIF 5 (double-blind).^10–13^ All the data were provided by AstraZeneca (AZ) data management at the same time. Due to file size, SPORTIF 3 was electronically transferred in 18 subsets. We analysed the data starting with the phase 2 studies and then progressed to phase 3 data to mimic the accrual of cases from a clinical development programme.

Records of subjects who were enrolled in countries that did not allow reuse of the trial data were excluded from the data set for regulatory concerns. Records of subjects who failed screening or withdrew consent during the trials were also excluded.

Data were fully de-identified, and this study was declared to be non-human subject research by the East Carolina University Institutional Review Board.^14^

### 2.2 | Parameters used for DILI-CAT phenotyping

Liver injury events were defined in line with FDA’s guidance for drug development^15^ as: (a) alanine aminotransferase (ALT) or aspartate aminotransferase (AST) ≥3 times (*) the upper limit of normal (ULN) or (b) alkaline phosphatase (ALP) or bilirubin ≥2*ULN. Three parameters were evaluated. The first parameter was latency, defined in days as the time between the start of the drug and time ALT or AST reached 3 × ULN or ALP reached 2 × ULN using the well-established formular. The other two parameters reflect liver injury pattern, specifically *R*-values were computed at the time ALT or AST reached 3 ULN or ALP reached 2 ULN using the well-established formular, ratio between "ALT in ULN" / "ALP in ULN",^8,9^ and AST/ALT ratio (de Ritis ratio)^16^ at onset of liver injury.

### 2.3 | Point allocation

Point allocations were chosen using our best judgement to assign points based on the importance of the variables used in the DILI-CAT. A full (100%) points were given in value for respective parameter when values of the specific case fell within the Interquartile range (IQR). Only half (50%) of the points available were given when values fell in the 15th-25th or 75th-85th percentile, and only 25% of the points available were given for values in the 10th-15th or 85th-90th percentile. Zero points were given when values fell in the 0th-10th or 90th-100th percentile. Points were deducted when values fell outside the range of the identified phenotype (a 25% reduction in points). If a value was both outside the range and an outlier, there was a 50% deduction in points (Table 2). Outliers were defined using a modified approach and described in supplement (Material S1).

### 2.4 | Steps for phenotyping and validating the ximelagatran-associated liver enzyme elevation

The fundamentals of the DILI-CAT approach are presented in abstract and manuscript that have been published in a preliminary form.^8,9^ In this setting of using DILI-CAT to reflect a drug development process, a stepwise approach was used, as in drug development, not all cases are available immediately.

In Step 1 (Table 1, see Figure S1 for a visual explanation), we identified a preliminary ximelagatran DILI-CAT phenotype using liver injury cases from the SPORTIF 2 clinical trial. The phenotype was developed based on the values for latency, *R*-value and AST/ALT ratio from cases reaching AST or ALT 3×ULN or AlkPhos 2×ULN. For each parameter (latency, *R*-value and AST/ALT ratio), we calculated median, interquartile range (IQR), percentile steps (0–10, 10–15 and 15–25 on either side of the IQR), and range as well as outlier values.

**FIGURE 1:**
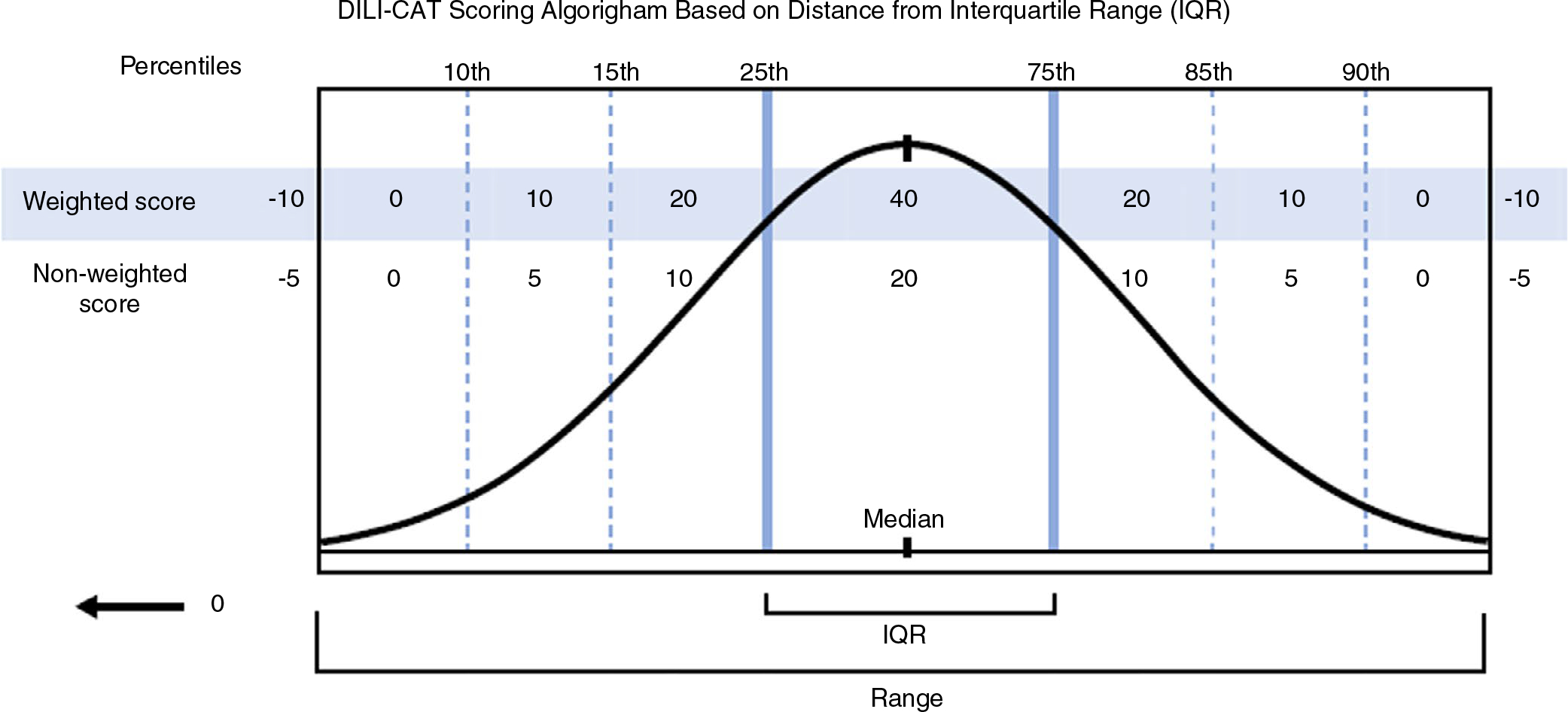
DILI-CAT scoring algorithm based on distance from interquartile range (IQR). Outliers definition: Values above IQR by 150% of the IQR width or below IQR by 75% of IQR width. Outliers get scored as minus 25%. If value is outside the range and an outlier, then it is scored minus 50%. DILI-CAT, drug-induced liver injury causality assessment tool

In Step 2, we assessed phenotype validity by comparing ximelagatran to warfarin cases. This was done by generating a score for each case using a scoring algorithm (Table 2). This algorithm was based on how closely cases matched the IQR for the working phenotype (Figure 1).

Step 3: The cumulative ximelagatran cases with elevated liver values from both data sets were then combined to develop a refined DILI-CAT phenotype (repeat Step 1).

Step 4: Then we repeated Step 2, which involved testing additional cases, again using the DILI-CAT scoring algorithm from the next even larger data set against the new refined phenotype. The concept is that these steps can be repeated as often as additional cases accrue from the trials in the clinical development programme, thereby leading to an ever more refined drug DILI-CAT phenotype along drug development.

### 2.5 | DILI-CAT scoring analysis and interpretation for ximelagatran-associated liver enzyme elevation

For each analysis, the parameter with the greatest statistical difference between ximelagatran and warfarin using Mantel-Haenszel rank sum test was considered to be the most important clinical feature distinguishing the two drugs and was therefore ‘weighted’ such that it could receive double the points of the other parameters–that is a maximum of 40 points (Table 2). The remaining two parameters could receive a maximum of 20 points. It was decided, a priori, that if there was no statistically significant difference among any of the parameters or no comparison data were available, latency would be used as the default ‘weighted’ parameter since time to onset of liver injury is typically an important element in the determination of DILI causality.^1–3,17,18^

### 2.6 | Statistical analysis

The nonparametric Mann-Whitney rank test was used to compare the ximelagatran phenotype to the warfarin phenotype for each of the three parameters, where a U-value of zero indicated lack of overlap–that is values from one drug are either all lower or all higher than the comparator. To assess the ability of the DILI-CAT-derived ximelagatran phenotype for differentiating liver injury events occurring during ximelagatran versus warfarin treatment, their respective DILI-CAT scores were tested for significance of difference using the Mantel–Haenszel test for trend, that considers the magnitude of ordinal values and therefore can be more powerful for numerical values compared to chi-square. In the event that two or more parameters had the same p value (using three decimal points), the U value was used to identify the most significant p value. Data handling was done using Microsoft Excel, and IBM SPSS version 25 was used for statistical analysis.

## 3 | RESULTS

### 3.1 | Clinical characteristics of the study populations

**FIGURE 2:**
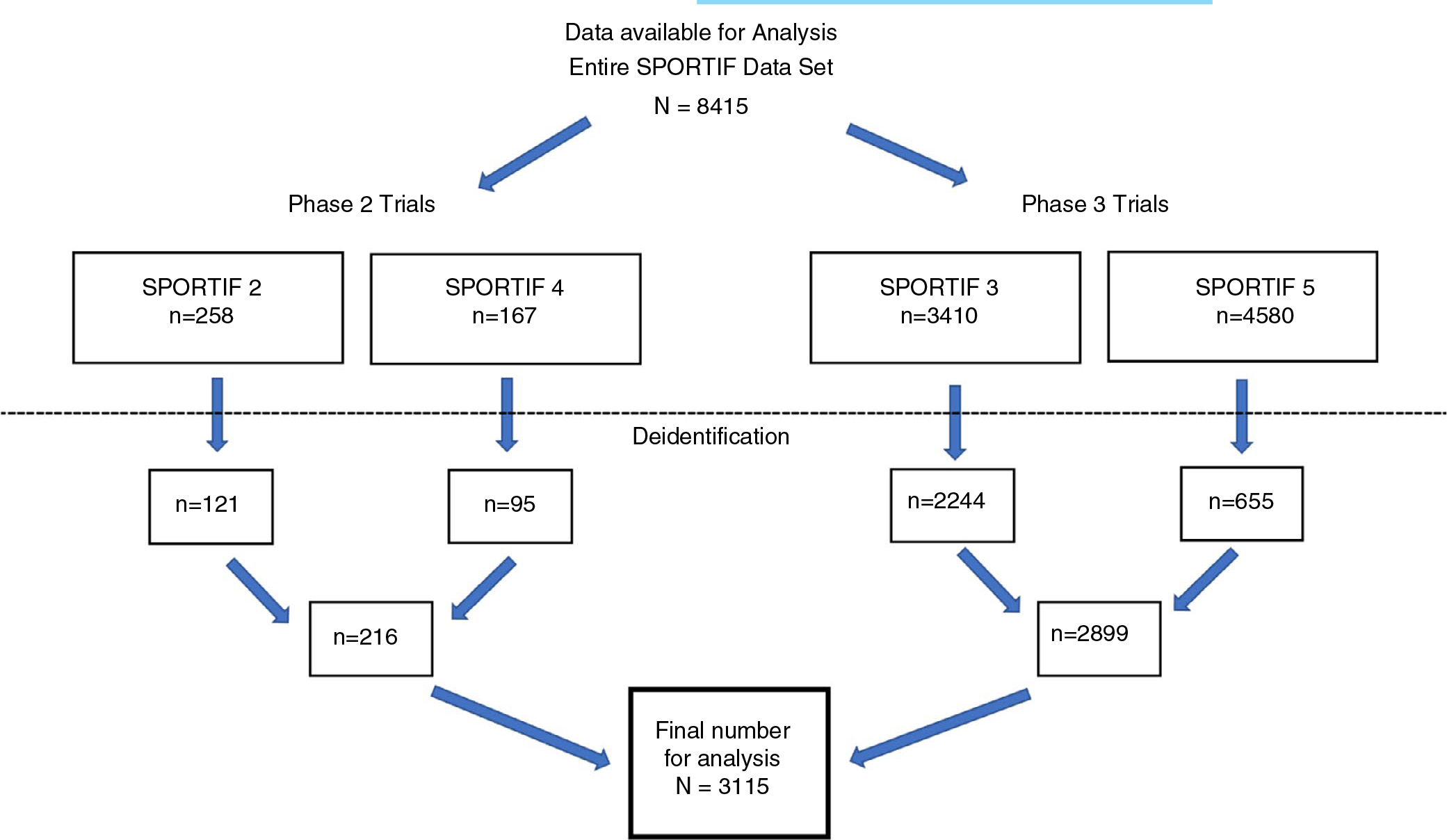
Data available for analysis, SPORTIF, stroke prevention using an ORal thrombin inhibitor in atrial fibrillation

Because of the rigorous de-identification process, basic demographic information such as age and race were not available. Data from 3115 out of 8415 subjects were available for analysis (Figure 2). Previous work by Lee et al,^19^ revealed that the same SPORTIF patient population had a mean duration of treatment with ximelagatran of 480 days and that the incidence of ALT >3*ULN was 7.9% in the ximelagatran group versus 1.2% in the comparator group.^19^ SPORTIF 2 treatment duration was limited to less than 90 days, while in the other SPORTIF studies treatments were extended to 3 years.

### 3.2 | Step 1. Preliminary DILI-CAT phenotyping for ximelagatran

The first data set was the phase 2 SPORTIF 2 trial with 116 subjects (86 on ximelagatran and 30 on warfarin). We identified five (9.3%) patients with liver injury in the ximelagatran arm and no patients with liver injury in the warfarin arm. All of the ximelagatran liver events occurred at visits six and seven, translating into a median latency of 69 days (IQR 58–78 days, Table 3). The median *R*-value was 8.7 (IQR 5.93–12.72), indicating hepatocellular injury, and the median AST/ALT ratio was 0.61 (IQR 0.60–0.65; Table 3).

### 3.3 | Step 2. Validating the preliminary DILI-CAT ximelagatran phenotype

Since we found no liver injury cases in the second small phase 2 data set (SPORTIF 4) to validate our preliminary DILI-CAT ximelagatran phenotype, we used one subset (out of a total 18 subsets) from the phase 3 SPORTIF 3 study. This data set of 191 subjects (86 on ximelagatran and 105 on warfarin) included eight patients with liver injury with five (5.8%) in the ximelagatran arm and three (2.9%) in the warfarin arm, this difference is not significant in chi-squared test (*p* = 0.3).

However, using the DILI-CAT concept, there was a statistically significant difference in the AST/ALT ratio among the groups (*p* = 0.025 Table 4), though *R*-value (*p* = 0.18) and latency (*p* = 0.764) were not significantly different for the 2 drugs (Tables 4 and 5). Thus, the DILI-CAT-S’s were calculated for individual cases with the AST/ALT ratio being the weighted parameter (ie double points for AST/ALT ratio ) since it showed the greatest difference among the parameters.

Applying the DILI-CAT scoring algorithm with phenotype derived from SPORTIF 2 to the SPORTIF 3 subset 1 cases demonstrated no significant difference in any single parameter alone using the individual DILI-CAT scores (Latency *p* = 0.46, *R*-value *p* = 0.13, and AST/ ALT ratio *p* = 0.069. However, when using the total DILI-CAT-S, the ximelagatran and warfarin cases were found to have significantly different clinical features (*p* = 0.016).

### 3.4 | Step 3—Refining the ximelagatran DILI-CAT phenotype

Next, we refined the ximelagatran phenotype by combining the original five cases of ximelagatran-associated liver injury from SPORTIF 2 with the additional five cases from SPORTIF 3 subset 1 (for a total of 10 ximelagatran-associated cases) to arrive at a refined DILI-CAT ximelagatran phenotype referred to here as the ximelagatran phenotype version 2 (Table 6).

### 3.5 | Step 4—validation of the ximelagatran phenotype version 2 (XPv2)

**FIGURE 3:**
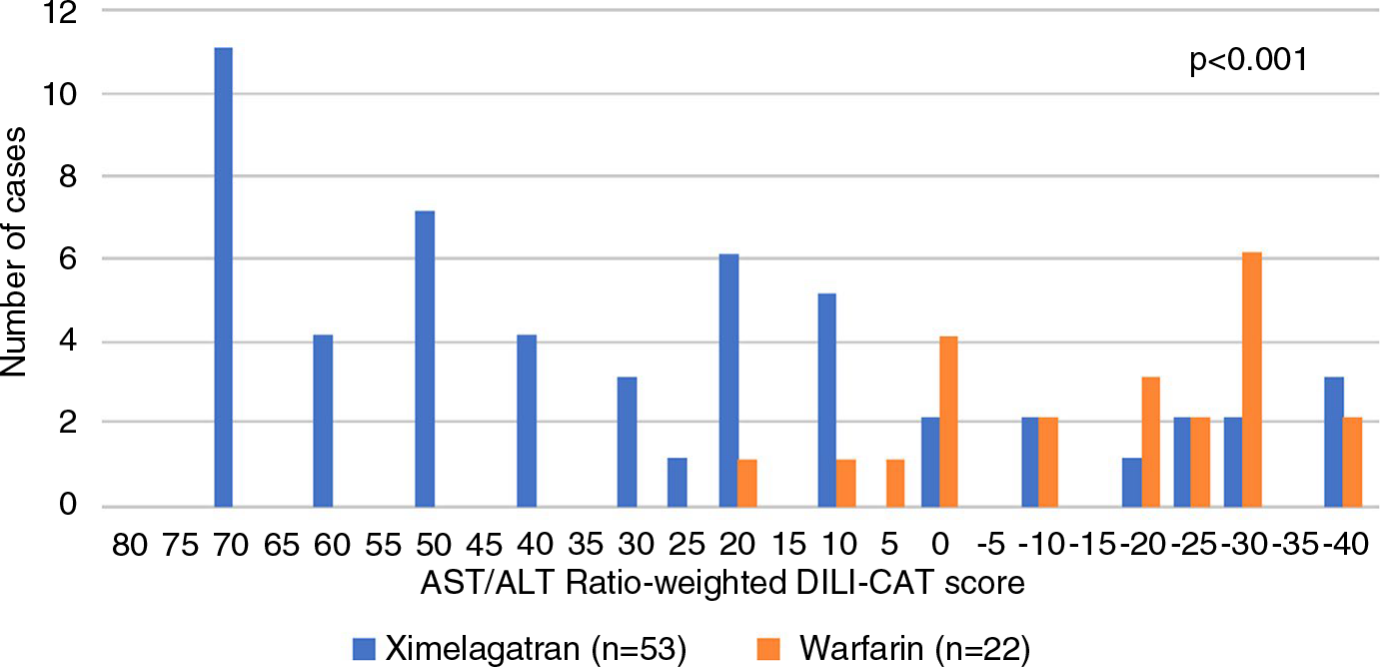
DILI-CAT scoring results for remaining SPORTIF 3 (subsets 2–18) plus SPORTIF 5 cases (using refined ximelagatran phenotype). ALT, alanine aminotransferase; AST, aspartate aminotransferase; DILI-CAT, drug-induced liver injury causality assessment tool; SPORTIF, stroke prevention using an ORal thrombin inhibitor in atrial fibrillation; XPv2, ximelagatran phenotype version 2

Next we applied the refined phenotype XPv2 to assess the remaining phase 3 data (subsets 2–18 from Sportif 3 and all cases from Sportif 3). A total of 2676 subjects (1343 on ximelagatran and 1333 on warfarin) made up the remaining SPORTIF 3 (17 subsets) plus the SPORTIF 5 data set. There were an additional 75 cases of liver injury including 53 (3.9%) ximelagatran cases and 22 (1.7%) warfarin cases (*p* < 0.001). Applying the DILI-CAT scoring algorithm to these 75 cases (see Table S1) and comparing the results to the XPv2 demonstrated that ximelagatran cases differ and show overall statistically significantly higher DILI-CAT-S’s (*p* < 0.001 for AST/ALT score, *p* = 0.001 for latency and *R*-value scores; Table 7, Figure 3).

### 3.6 | Iterative refining of the phenotype

A third and final iteration of re-refined phenotype resulted from combining all of the cases from the entire SPORTIF data set to develop the most updated refined ximelagatran phenotype (XPv3). This included 73 cases of liver injury in the ximelagatran arms and 28 cases in the warfarin arms (Table 8).

## 4 | DISCUSSION

This study provides compelling evidence that the DILI-CAT offers an objective, accessible and reproducible approach to mitigate some of the inter- and intra-rater variability of DILI causality assessment. Underpinned by clinical knowledge and experience, DILI-CAT defines a drug’s phenotype by allotting points based on a defined computer-assisted yet straightforward algorithm instead of a manual instrument where human interpretation can lead to variation in scores.^3,17^ DILI-CAT fills the need for a computer-assisted method for DILI causality assessment as previously suggested.^17^

Of note, this model was highly predictive even in the absence of formal adjudication results of the cases; and where most of the severe cases of DILI fulfilling Hy’s Law criteria (ie ALT or AST ≥3× ULN and total bilirubin >2× ULN) had been removed from the database available for our analysis due to privacy concerns. However, these adjudication data have been presented previously by Lee et al, which included some patient-level data in their paper on 42 patients with or without an alternative identifiable cause for liver injury.^19^ Thirty-seven of those 42 (88%) patients were exposed to ximelagatran. Nineteen of the 37 (51%) had alternative diagnoses for elevated liver enzymes, while 18 out of the 37 (49%) had no alternative diagnosis other than DILI. *R*-value and AST/ALT ratio were unavailable for the cases, but latency was published. We analysed this latency data using the DILI-CAT scoring algorithm by (a) presence or absence of an alternative diagnosis for patients on ximelagatran and (b) treatment group (ximelagatran vs. not on ximelagatran) for cases without an alternative diagnosis (Figure S2). For subjects on ximelagatran, results showed a statistically significant difference in liver injury cases when comparing those with versus those without an alternative diagnosis (*p* = 0.013). No significant difference was found between those with liver injury on ximelagatran without alternative diagnosis versus those not on ximelagatran (*p* = 0.073), although nominally it approached statistical significance, despite the small number not on Ximelagatran (*n* = 5).

It is possible that the DILI-CAT score generated is unable to differentiate clinical features of two or more drugs. For instance, when comparing two specific drugs, it is possible that the DILI phenotype for both drugs is so similar that it would require many more cases to detect a difference (assuming a difference exists). It could also be that one of the two drugs has such a wide range of variability for one of the parameters that it creates an overlapping phenotype with the other drug. In case of concomitantly administered medications, drugs that have a synergistic or an additive effect, and are therefore co-dependent, would make the combination signature different from the monotherapy signature. This latter possibility would theoretically allow exploration of different phenotype profiles for monotherapy versus combination drug regiments.

Given the high cost of drug development and, more importantly, the potential risk to patients, clinical research must be able to detect potential hepatotoxicity as early and as reliably as possible in the drug development process.^20^ The best practice for hepatotoxicity causality assessment remains expert opinion.^4–6^ Unfortunately, while robust, it is not practical to attain expert opinion for every case of liver injury in routine clinical practice. Although ximelagatran was granted marketing approval in several countries, the US FDA did not grant approval because, in part, of concerns over potential hepatotoxic effects of the drug suggested by elevations of alanine aminotransferase. Indeed, Southworth et al in 2014 published results of a post hoc analysis of the SPORTIF studies using extreme value modelling and found evidence of a liver signal in the phase 2 data, concluding that development could have been halted before further phase 3 development if such statistical methodology had been available at that time.^21^ This would have been further strengthened by additional adjudication support tool such as DILI-CAT.

We recognise limitations of this study. First, limited patient level data were available from the SPORTIF programmes. However, the data captured were carefully curated, and complete liver tests along with other critical variables, including timing of the drug and injury, were available. Additionally, the DILI-CAT version presented here does not incorporate potentially important clinical elements such as concomitant medications. Hence, drug-drug and drug–host interaction cannot be fully assessed; this could be important since it is possible that underlying NAFLD may increase the risk for DILI,^22^ and female gender may be associated with a hepatocellular presentation of DILI.^23^ Nonetheless, further study is required to better understand whether host factors alter the drug-specific phenotypes. Also, data on comorbidities, especially those leading to hepatic impairment, are not captured in this model. However, these factors are somewhat mitigated by the routine process of screening study subjects with established inclusion and exclusion criteria. This results in a general effect of decreasing heterogeneity. Theoretically, the more homogeneous the population, the more similar one might expect the patterns to be for latency and biochemical features in bona fide DILI cases due to the implicated drug. De-challenge and re-challenge timing data may also improve the predictive ability of this method. Unfortunately, such data are often not accurately and consistently captured in clinical trial or real-world settings. In this study, the de-challenge parameter was not included since complete information was not always available in the data set. Finally, given that the premise of DILI-CAT is based on fundamental clinical criteria such as time to onset (ie latency) and biomarkers of pathophysiology (ie biochemical features analogous to *R*-value and AST/ALT ratio), the door is open for further exploration into applying these same principles to other organ-specific drug-induced adverse events. These would include other gastrointestinal, dermatologic, renal, cardiac, haematologic or central nervous system adverse events, to name a few. Ideally, any identified case of concern would also be confirmed in in vitro assays as monocytes derived hepatocyte like cells.^24^ In a number of studies, this test has shown promise as a confirmatory study.^25,26^

In addition, the most severe cases of DILI were not available for this study due to data privacy concerns. However, if they had been available, this data conceivably would have improved the discrimination potential of the DILI-CAT. Any potential effects of age or race on this model remain unknown as these variables were removed in order to anonymise the data, but they could offer opportunities for further research and refinement of this causality assessment methodology.

In conclusion, we have presented a model that may help to detect an early DILI signal by identifying events in relation to a clinical phenotype (timing and biochemical pattern). Although formal reproducibility and validity testing has not yet been performed, this analysis suggests that the DILI-CAT provides a consistent and efficient way to distinguish between cases where the drug is responsible for DILI and cases where it is not and may be applicable across different stages of clinical development. In the future, prospective inclusion of an effort to detect (early) DILI events using the DILI-CAT approach may prove to identify DILI signals early and improve overall drug development.

## Supplementary Material

supinfo

## Figures and Tables

**TABLE 1:** DILI-CAT step sequence, ximelagatran phenotype

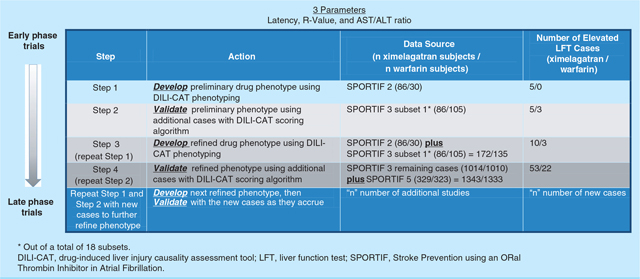

Abbreviations: DILI-CAT, drug-induced liver injury causality assessment tool; LFT, liver function test; SPORTIF, Stroke Prevention using an ORal Thrombin Inhibitor in Atrial Fibrillation.

“n” reflective of number of additional cases becoming available.

a Out of a total of 18 subsets.

**TABLE 2:** DILI-CAT scoring algorithm

Within	Percentage of points per parameter	Final score for the standard parameters	Final score for the “weighted” parameter
IQR (25th-75th percentile)	100	20	40
25th-15th percentile	50	10	20
75th-85th percentile			
15th-10th percentile	25	5	10
85th-90th percentile			
10th percentile to minimum of IQR	0	0	0
90th percentile to maximum of range			
Above/below upper/lower range bounds^a^	−25	−5	−10
Outlier^a^	−25	−10	−10

Abbreviations: DILI-CAT, drug-induced liver injury causality assessment tool; IQR, interquartile range.

a If a value is both outside the range and an outlier, −50% is used.

**TABLE 3:** Preliminary DILI-CAT phenotype of ximelagatran based on five cases identified in SPORTIF 2 which was limited to 90 day treatment. (a) shows individual data, (b) shows the median, IQR, percentile, range and outlier values

**Patient ID**	**Day of therapy**	***R*-value**	**AST/ALT ratio**
(a) Individual data from cases with elevated liver enzymes from SPORTIF 2			
Ximelagatran case 2.1	69	12.7	0.49
Ximelagatran case 2.2	83	8.7	0.65
Ximelagatran case 2.3	56	5.9	0.61
Ximelagatran case 2.4	58	5.8	0.6
Ximelagatran case 2.5	75	13.9	0.69
	**Latency (days)**	***R*-value**	**AST/ALT ratio**
(b) Statistics for cases with elevated liver enzymes from SPORTIF 2			

Median	69	8.68	0.61
IQR low	58	5.93	0.6
IQR high	75	12.72	0.65
15th-25th percentile	57.2	5.86	0.56
75th-85th percentile	78.2	13.12	0.67
10th-15th percentile	56.8	5.82	0.53
85th-90th percentile	79.8	13.4	0.67
Outlier low	45.3	0.84	0.53
Outlier high	100.5	22.9	0.73
Range	56–83	5.75–13.85	0.49–0.69

Abbreviations: ALT, alanine aminotransferase; AST, aspartate aminotransferase; DILI-CAT, drug-induced liver injury causality assessment tool; IQR, interquartile range.

**TABLE 4:** DILI-CAT scores for SPORTIF 3 (subset 1) cases using the preliminary DILI-CAT ximelagatran phenotype (derived from SPORTIF 2)

Treatment arm	Latency	*R*-value	AST/ALT ratio	Latency Score	*R*-value score	AST/ALT ratio score^a^	Total score (AST/ALT ratio weighted)
Ximelagatran case 1	43	3.32	0.64	−10	−5	20 (40)	25
Ximelagatran case 2	46	6.63	0.68	−5	20	0 (0)	15
Ximelagatran case 3	92	7.26	0.59	−5	20	10 (20)	35
Ximelagatran case 4	427	9.41	0.47	−10	20	−10 (−20)	−10
Ximelagatran Case 5	45	0.56	0.65	−10	−10	20 (40)	20
Warfarin case 1	43	2.07	1.11	−10	−5	−10 (−20)	−35
Warfarin case 2	187	2.82	0.97	−10	−5	−10 (−20)	−35
Warfarin case 3	266	0.67	1.09	−10	−5	−10 (−20)	−40

*Note:* Dark green = values fitting within the IQR of the phenotype. Light green = light green indicates values that are between the 10th and 25th or between the 75th and 90th percentile. White = values outside of IQR, but still within the range. Light red = outside of range but not outlier. Red = outside of range AND outlier.

Abbreviations: ALT, alanine aminotransferase; AST, aspartate aminotransferase; DILI-CAT, drug-induced liver injury causality assessment tool; SPORTIF, Stroke Prevention using an ORal Thrombin Inhibitor in Atrial Fibrillation.

a In this case, the AST/ALT ratio showed the greatest potential to discern ximelagatran from warfarin, so the points for this parameter were counted as double value.

**TABLE 5:** Summary statistics for DILI-CAT scores for SPORTIF 3 (subset 1) using the preliminary DILI-CAT ximelagatran phenotype (derived from SPORTIF 2)

Treatment	Latency	*R*-value	AST/ALT ratio	Latency score	*R*-value score	AST/ALT ratio score	Total score (AST/ALT ratio weighted)^a^
Statistics for original values (Tables 4 )							
Ximelagatran (*n* = 5) median	46	6.63	0.64	NA	NA	NA	NA
Warfarin (*n* = 3) median	187	2.07	1.09	NA	NA	NA	NA
Mann–Whitney *U* value	6.5	3.0	0.0^b^	NA	NA	NA	NA
Mann–Whitney rank test, *p*-value	0.764	0.18	0.025^b^	NA	NA	NA	NA
Statistics for distance from IQR and for DILI-CAT score and DILI-CAT scores for latency, *R*-value, AST/ALT ratio and total score							
Ximelagatran median distance from IQR	15	0.0	0.01	−10	20	10 (20)^a^	30
Warfarin median distance from IQR	112	3.86	0.44	−10	−5	−10 (−20)^a^	−35
Mann–Whitney *U* value for distance from IQR	4–5	3	0^b^	4.5	3.5	1.5	NA
Mann–Whitney test for distance from IQR *p*-value	0.368	0.177	0.024^b^	0.393	0.25	0.071^b^	0.036^b^
Mantel–Haenszel test for trend	NA	NA	NA	0.464	0.129	0.069^b^	0.016^b^

*Note:* The darker grey shaded area indicates the Mann–Whitney test for statistical differences.

Abbreviations: ALT, alanine aminotransferase; AST, aspartate aminotransferase; DILI-CAT, drug-induced liver injury causality assessment tool; IQR, interquartile range; NA, not applicable; SPORTIF, Stroke Prevention using an ORal Thrombin Inhibitor in Atrial Fibrillation.

a double points for AST/ALT ratio due to showing the greatest difference between ximelagatran and warfarin in rank sum test.

b Significant differences in Mann–Whitney test for distance, and for the most marked difference defined by lowest Mann-Whitney *U*-value.

**TABLE 6:** Ximelagatran phenotype version 2 (SPORTIF 2 plus SPORTIF 3, subset 1)

	Latency (days)	*R*-value	AST/ALT ratio
Median	6.5	6.95	0.625
IQR low	45.75	5.14	0.565
IQR high	85.25	10.24	0.658
15th-25th percentile	44.3	2.35	0.483
75th-85th percentile	209.25	13.12	0.684
10th-15th percentile	43.2	0.84	0.472
85th-90th percentile	393.5	13.74	0.689
Outlier low	16.125	1.32	0.496
Outlier high	144.5	17.88	0.796
Range	43–427	0.56–18.85	0.47–0.69

Abbreviations: ALT, alanine aminotransferase; AST, aspartate aminotransferase; IQR, interquartile range; SPORTIF, Stroke Prevention using an ORal Thrombin Inhibitor in Atrial Fibrillation.

**TABLE 7:** DILI-CAT scoring algorithm applied to the remaining SPORTIF 3 (subsets 2–18) plus SPORTIF 5 cases using the XPv2 (derived from SPORTIF 2 plus SPORTIF 3 subset 1)

Treatment	Latency	*R*-value	AST/ALT ratio	Latency score	*R*-value score	AST/ALT score	Total score (AST/ALT ratio weighted)^a^
Statistics for original values							
Ximelagatran (*n* = 53) median	88	4.43	0.65	NA	NA	NA	NA
Warfarin (*n* = 22) median	155	1.05	1.136	NA	NA	NA	NA
Mann–Whitney *U* value	328	351	310^a^	NA	NA	NA	NA
Mann–Whitney rank test *P*-value	0.003	0.007	0.001^a^	NA	NA	NA	NA
Statistics for distance from IQR and for DILI-CAT							
Ximelagatran *(n* = 53) median distance from IQR	2.75	0.81	0.065	10	10	0 (0)^a^	20
Warfarin *(n* = 22) median distance from IQR	69.75	4.09	0.478	0	0	−10 (−20)^a^	−20
Mann–Whitney U for distance from IQR	252	350	199.5^b^	286	350.5	211.5	181
Mann–Whitney test for distance from IQR *P*-value	<0.001	0.004	<0.001	<0.001	0.004	<0.001	<0.001
DILI-CAT assessed by Mantel-Haenszel test for trend	NA	NA	NA	0.001^c^	0.001^c^	<0.001^c^	<0.001^c^

Abbreviations: ALT, alanine aminotransferase; AST, aspartate aminotransferase; DILI-CAT, drug-induced liver injury causality assessment tool; IQR, interquartile range; NA, not applicable; SPORTIF, Stroke Prevention using an ORal Thrombin Inhibitor in Atrial Fibrillation; XPv2, ximelagatran phenotype version 2.

a Lowest *U* value, therefore most significant.

b Significant differences in Mann–Whitney test for distance, and for the most marked difference defined by lowest Mann–Whitney *U* value; the darker grey shaded area indicates the Mann–Whitney test for statistical differences.

c significant differences in Mantel-Henszel Test

**TABLE 8:** Ximelagatran phenotype version 3

	Latency (days)	*R*-value	AST/ALT ratio
Median	86	4.7	0.642
IQR low	62	3.21	0.567
IQR high	106	6.45	0.8
15th-25th Percentile	58.3	0.74	0.542
75th-85th Percentile	175.6	8.74	0.995
10th-15th Percentile	56.3	0.52	0.504
85th-90th Percentile	278.8	8.98	1.42
Outlier low	29	0.78	0.393
Outlier high	17.3	11.33	1.149
Range	43–427	0.06–13.85	0.47–6.43

Abbreviations: ALT, alanine aminotransferase; AST, aspartate aminotransferase; IQR, interquartile range.

## Data Availability

All data used are own by AstraZeneca, who would need to be contacted for data access.

## Key Points

- Currently available drug-induced liver injury (DILI) adjudication scoring systems treat all drugs similarly.
- We utilised a novel data-driven computerised algorithmic causality tool known as DILI-CAT (DILI causality assessment tool), which aims to characterise a specific DILI phenotype for specific drugs–in this case using ximelagatran as an example.
- This approach clearly led to identification of a DILI signal for ximelagatran, and theoretically could have been used during early drug development to highlight ximelagatran’s potential hepatotoxicity phenotype.
