# Supplementary material for: A novel phenotype-based drug-induced liver injury causality assessment tool (DILI-CAT) allows for signal confirmation in early drug development
Source: Aliment Pharmacol Ther. Author manuscript; Available in PMC 2023 Apr 1. (PMC9164935; doi:10.1111/apt.16836)
Supplement: supinfo [file NIHMS1795202-supplement-supinfo.docx]

**Supplementary Material**

**Supplemental Figure 1: Overview of the step wise approach**

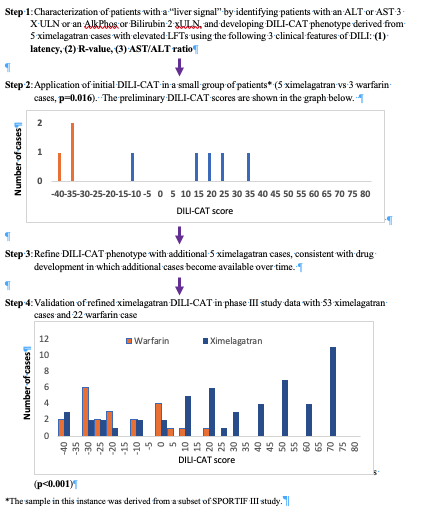


Outlier definition:

Outlier definition was modified from Moore & McCabe. Outlier values above the upper bound of the interquartile range (IQR) were defined as those that were at least 150% of the absolute difference between the upper and lower bounds of the IQR. Given the skewed nature of the data toward zero, outlier values below the lower bound of the IQR were defined as those that were at least 75% of the absolute difference, thereby reducing the chance of a lower outlier range extending into negative (clinically nonsensical) numbers.

Moore DS, McCabe GP. Introduction to the Practice of Statistics, 3rd ed. New York: W. H. Freeman, 1999.

**Supplemental Figure 2. DILI-CAT latency scoring of Lee et al. adjudicated cases using ximelagatran phenotype version 3**

**
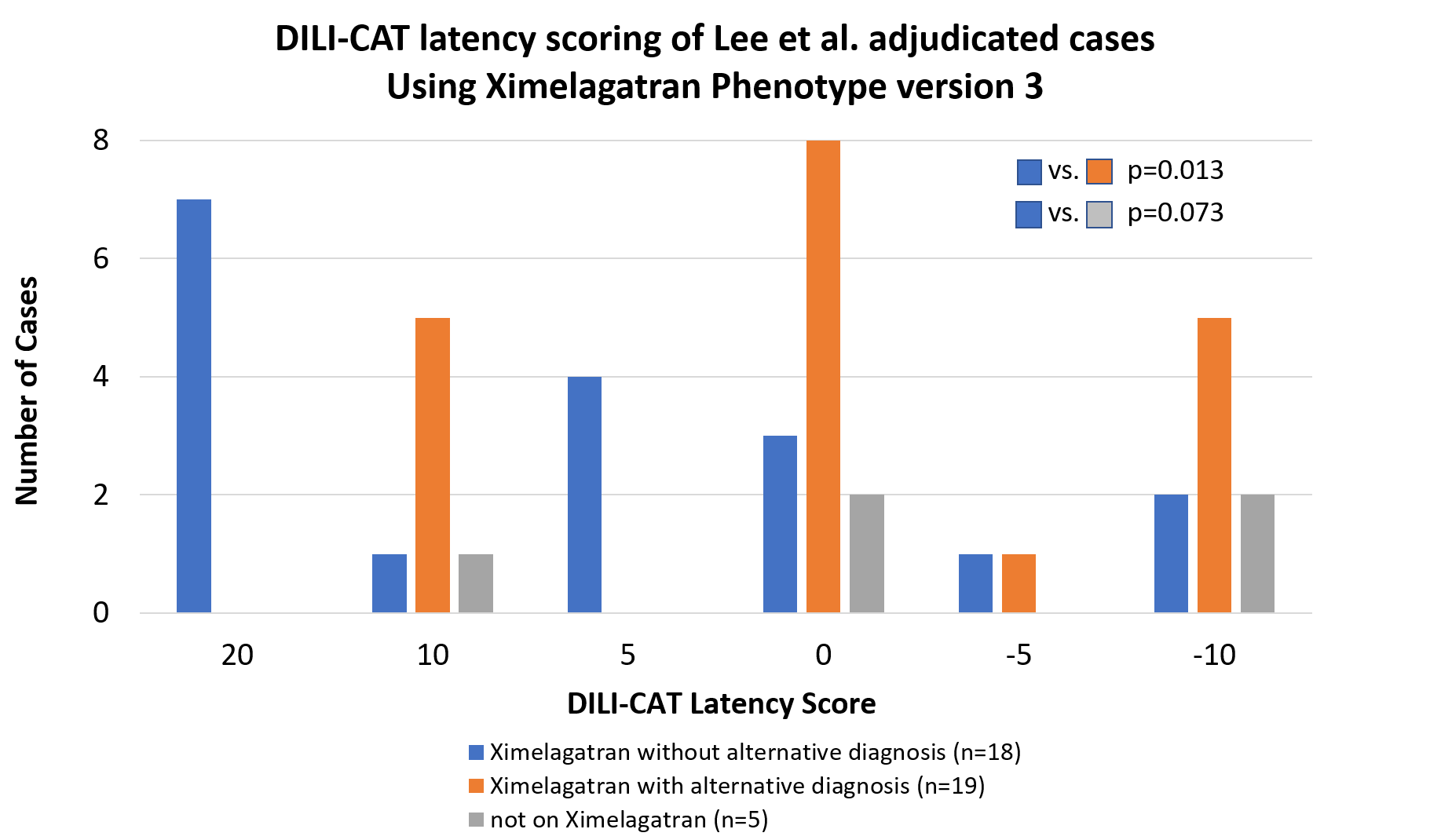
**

DILI-CAT, drug-induced liver injury causality assessment tool.

**Supplemental Table 1. DILI-CAT scores for SPORTIF 3 (subsets 2-18) and SPORTIF 5 cases using the refined DILI-CAT ximelagatran phenotype**

| **Treatment** | **Latency** | **R-value** | **AST/ALT ratio** | **Latency score** | **R-value score** | **AST/ALT ratio score*** | **Total score (AST/ALT ratio weighted)** | |
| --- | --- | --- | --- | --- | --- | --- | --- | --- |
| Ximelagatran | 32 | 0·52 | 0·61 | -5 | -10 | 20 (40) | 25 |  |
| Ximelagatran | 51 | 0·06 | 6·43 | 20 | -10 | -10 (-20) | -10 |  |
| Ximelagatran | 56 | 5·70 | 0·68 | 20 | 10 | 5 (10) | 40 |  |
| Ximelagatran | 58 | 3·09 | 0·73 | 20 | 0 | -5 (-10) | 10 |  |
| Ximelagatran | 58 | 3·21 | 0·67 | 20 | 0 | 5 (10) | 30 |  |
| Ximelagatran | 60 | 4·66 | 0·52 | 20 | 10 | 0 | 30 |  |
| Ximelagatran | 60 | 3·23 | 0·72 | 20 | 5 | -5 (-10) | 15 |  |
| Ximelagatran | 61 | 9·44 | 0·47 | 20 | 10 | -5 (-10) | 20 |  |
| Ximelagatran | 62 | 7·62 | 0·50 | 20 | 20 | 0 | 40 |  |
| Ximelagatran | 62 | 9·98 | 0·77 | 20 | 10 | -10 (-20) | 10 |  |
| Ximelagatran | 62 | 5·09 | 0·64 | 20 | 10 | 20 (40) | 70 |  |
| Ximelagatran | 64 | 4·18 | 0·66 | 20 | 10 | 10 (20) | 50 |  |
| Ximelagatran | 74 | 2·92 | 0·89 | 20 | -5 | -10 (-20) | -5 |  |
| Ximelagatran | 80 | 6·46 | 0·49 | 20 | 20 | 0 | 40 |  |
| Ximelagatran | 83 | 3·65 | 0·57 | 20 | 5 | 10 (20) | 45 |  |
| Ximelagatran | 85 | 3·49 | 0·81 | 20 | 5 | -10 (-20) | 5 |  |
| Ximelagatran | 85 | 4·70 | 0·76 | 20 | 10 | -10 (-20) | 10 |  |
| Ximelagatran | 86 | 5·44 | 0·49 | 10 | 10 | -5 (-10) | 10 |  |
| Ximelagatran | 87 | 3·56 | 0·60 | 10 | 5 | 20 (40) | 55 |  |
| Ximelagatran | 87 | 5·91 | 0·55 | 10 | 20 | 0 | 30 |  |
| Ximelagatran | 88 | 8·96 | 0·54 | 10 | 20 | 0 | 30 |  |
| Ximelagatran | 88 | 3·27 | 0·78 | 10 | 5 | -10 (-20) | -5 |  |
| Ximelagatran | 92 | 8·98 | 0·53 | 5 | 20 | 0 | 25 |  |
| Ximelagatran | 92 | 4·77 | 0·64 | 5 | 10 | 20 (40) | 55 |  |
| Ximelagatran | 93 | 3·54 | 0·54 | 5 | 5 | 0 | 10 |  |
| Ximelagatran | 93 | 6·24 | 0·62 | 5 | 20 | 20 (40) | 65 |  |
| Ximelagatran | 93 | 4·08 | 0·96 | 5 | 5 | -10 (-20) | -10 |  |
| Ximelagatran | 96 | 8·83 | 0·59 | 5 | 20 | 10 (20) | 45 |  |
| Ximelagatran | 106 | 5·87 | 0·57 | 5 | 20 | 10 (20) | 45 |  |
| Ximelagatran | 117 | 6·92 | 0·57 | 5 | 20 | 10 (20) | 45 |  |
| Ximelagatran | 149 | 0·41 | 1·63 | -5 | -10 | -10 (-20) | -35 |  |
| Ximelagatran | 178 | 5·02 | 0·65 | -5 | 10 | 20 (40) | 45 |  |
| Ximelagatran | 181 | 5·39 | 0·69 | -5 | 10 | -5 (-10) | -5 |  |
| Ximelagatran | 182 | 0·83 | 1·66 | -5 | -5 | -10 (-20) | -30 |  |
| Ximelagatran | 303 | 0·12 | 1·46 | -5 | -10 | -10 (-20) | -35 |  |
| Ximelagatran | 309 | 0·70 | 0·93 | -5 | -5 | -10 (-20) | -30 |  |
| Ximelagatran | 314 | 12·64 | 0·55 | -5 | 5 | 0 | 0 |  |
| Ximelagatran | 552 | 0·55 | 1·38 | -10 | -10 | -10 (-20) | -40 |  |
| Ximelagatran | 637 | 0·33 | 1·81 | -10 | -10 | -10 (-20) | -40 |  |
| Ximelagatran | 59 | 4·87 | 0·47 | 20 | 10 | -5 (-10) | 20 |  |
| Ximelagatran | 59 | 4·43 | 1·01 | 20 | 10 | -10 (-20) | 10 |  |
| Ximelagatran | 62 | 4·33 | 0·73 | 20 | 10 | -5 (-10) | 20 |  |
| Ximelagatran | 62 | 2·92 | 0·58 | 20 | -5 | 10 (20) | 35 |  |
| Ximelagatran | 64 | 5·45 | 1·43 | 20 | 10 | -10 (-20) | 10 |  |
| Ximelagatran | 72 | 2·59 | 0·62 | 20 | -5 | 20 (40) | 55 |  |
| Ximelagatran | 91 | 0·45 | 1·31 | 5 | -10 | -10 (-20) | -25 |  |
| Ximelagatran | 93 | 5·56 | 0·62 | 5 | 10 | 20 (40) | 55 |  |
| Ximelagatran | 99 | 6·96 | 0·59 | 5 | 20 | 10 (20) | 45 |  |
| Ximelagatran | 102 | 4·6 | 0·8 | 5 | 10 | -10 (-20) | -5 |  |
| Ximelagatran | 153 | 3·37 | 0·85 | -5 | 5 | -10 (-20) | -20 |  |
| Ximelagatran | 155 | 3·92 | 0·58 | -5 | 5 | 10 (20) | 20 |  |
| Ximelagatran | 170 | 5·97 | 0·56 | -5 | 20 | 10 (20) | 35 |  |
| Ximelagatran | 560 | 0·44 | 1·88 | -10 | -10 | -10 (-20) | -40 |  |
| Ximelagatran median | **88** | **4·43** | **0·65** | **10** | **10** | **0** | **20** |  |
| Warfarin | 69 | 7·54 | 0·43 | 20 | 20 | -10 (-20) | 20 |  |
| Warfarin | 92 | 5·00 | 1·00 | 5 | 10 | -10 (-20) | -5 |  |
| Warfarin | 118 | 3·44 | 0·99 | 5 | 5 | -10 (-20) | -10 |  |
| Warfarin | 123 | 0·51 | 1·07 | 5 | -10 | -10 (-20) | -25 |  |
| Warfarin | 126 | 2·57 | 0·36 | 0 | -5 | -10 (-20) | -25 |  |
| Warfarin | 146 | 1·17 | 1·53 | -5 | -5 | -10 (-20) | -30 |  |
| Warfarin | 148 | 0·74 | 1·50 | -5 | -5 | -10 (-20) | -30 |  |
| Warfarin | 155 | 0·29 | 1·39 | -5 | -10 | -10 (-20) | -35 |  |
| Warfarin | 155 | 0·45 | 1·38 | -5 | -10 | -10 (-20) | -35 |  |
| Warfarin | 186 | 4·44 | 1·49 | -5 | 10 | -10 (-20) | -15 |  |
| Warfarin | 306 | 0·18 | 1·59 | -5 | -10 | -10 (-20) | -35 |  |
| Warfarin | 307 | 0·38 | 2·32 | -5 | -10 | -10 (-20) | -35 |  |
| Warfarin | 365 | 5·03 | 1·24 | -5 | 10 | -10 (-20) | -15 |  |
| Warfarin | 370 | 7·03 | 1·46 | -5 | 20 | -10 (-20) | -5 |  |
| Warfarin | 460 | 0·34 | 0·72 | -10 | -10 | -5 (-10) | -30 |  |
| Warfarin | 462 | 0·29 | 1·18 | -10 | -10 | -10 (-20) | -40 |  |
| Warfarin | 568 | 1·01 | 1·00 | -10 | -5 | -10 (-20) | -35 |  |
| Warfarin | 691 | 0·43 | 1·73 | -10 | -10 | -10 (-20) | -40 |  |
| Warfarin | 32 | 0·9 | 1·06 | -5 | -5 | -10 (-20) | -30 |  |
| Warfarin | 48 | 1·54 | 1·09 | 20 | -5 | -10 (-20) | -5 |  |
| Warfarin | 61 | 12·38 | 0·38 | 20 | 5 | -10 (-20) | 5 |  |
| Warfarin | 191 | 1·09 | 1·09 | -5 | -5 | -10 (-20) | -30 |  |
| Warfarin median | **155** | **1·05** | **1·14** | **-5** | **-5** | **-10** | **-30** |  |

* In this case, the AST/ALT ratio showed the greatest potential to discern ximelagatran from warfarin, so the points for this parameter were counted as double value.

Dark green = values fitting within the IQR of the phenotype.

Light green = values that are between the 10^th^ and 25^th^ or between the 75^th^ and 90^th^ percentile.

White = values outside of IQR, but still within the range.

Light red = outside of range but not outlier.

Red = outside of range AND outlier.

ALT, alanine aminotransferase; AST, aspartate aminotransferase; DILI-CAT, drug-induced liver injury causality assessment tool; SPORTIF, Stroke Prevention using an ORal Thrombin Inhibitor in Atrial Fibrillation.
